# Surgical Outcomes of Laparoscopic Pelvic Lymph Node Debulking during Staging Aortic Lymphadenectomy in Locally Advanced Cervical Cancer: A Multicenter Study

**DOI:** 10.3390/cancers14081974

**Published:** 2022-04-13

**Authors:** Berta Díaz-Feijoó, Úrsula Acosta, Aureli Torné, Blanca Gil-Ibáñez, Alicia Hernández, Santiago Domingo, Melissa Bradbury, Antonio Gil-Moreno

**Affiliations:** 1 Gynecology Oncology Unit, Institute Clinic of Gynecology, Obstetrics and Neonatology, Hospital Clínic de Barcelona, 08036 Barcelona, Spain; atorne@clinic.cat; 2 Institut d ’Investigacions Biomèdiques August Pi i Sunyer (IDIBAPS), Faculty of Medicine, University of Barcelona, 08036 Barcelona, Spain; 3 Service of Gynecology Oncology, Hospital Universitari Vall d’Hebron, Universitat Autònoma de Barcelona, 08035 Barcelona, Spain; uacosta@vhebron.net (Ú.A.); mbradburylobato@gmail.com (M.B.); agil@vhebron.net (A.G.-M.); 4 Department of Obstetrics and Gynecology, Hospital Universitario 12 de Octubre, 28041 Madrid, Spain; blancalabacin@hotmail.com; 5 Department of Gynecology, Hospital Universitario La Paz, 28046 Madrid, Spain; aliciahernandezg@gmail.com; 6 Department of Gynecology Oncology, Hospital Universitario La Fe, 46026 Valencia, Spain; santiagodomingodelpozo@gmail.com; 7 Centro de Investigación Biomédica en Red de Cáncer, CIBERONC, 28041 Madrid, Spain

**Keywords:** aortic lymphadenectomy, locally advanced cervical cancer, pelvic lymph node debulking, surgical staging

## Abstract

**Simple Summary:**

Lymph node metastasis is an important prognostic factor in locally advanced cervical cancer (LACC), which makes correct staging crucial. In contrast to existing studies evaluating pelvic lymphadenectomy after aortic lymphadenectomy, this study focuses on the pelvic node (PLN) debulking technique which has the dual objective of staging and cytoreduction. This is a multicenter retrospective study of patients with LACC and positive pelvic nodes on imaging tests. Feasibility, morbidity and delay in the initiation of chemoradiotherapy (CRT) were evaluated for the PLN debulking by comparing it with a control group of aortic lymphadenectomy alone. Excision of the bulky nodes was possible in 99.4% of patients. There were no differences in complications between the groups and a shorter time from diagnosis and from surgery to the start of CRT was observed in the study group.

**Abstract:**

Background: Few studies have evaluated laparoscopic pelvic lymph node (PLN) debulking during staging aortic lymphadenectomy in locally advanced cervical cancer (LACC). It allows us to know the lymph node status and facilitates the action of chemoradiotherapy (CRT) by reducing tumor burden. We evaluated its feasibility and compared the perioperative morbidity and the time to CRT with a control group. Methods: This was a multicenter retrospective study of patients with LACC FIGO stage IIIC1r who were recipients of CRT. We compared two cohorts: group 1, which consisted of 164 patients with surgical staging by laparoscopic aortic lymphadenectomy and PLN debulking, and group 2, which consisted of 111 patients with aortic lymphadenectomy alone. Results: Excision of the bulky nodes was possible in all patients in group 1 except for one. Surgery lasted a median of 82 min longer in group 1 but there was no greater intraoperative bleeding or increased hospital stay. There were no significant differences in intraoperative or postoperative complications between the groups. A significantly shorter time from surgery to the start of RT was observed in group 1. Conclusions: It is feasible to perform laparoscopic PLN debulking in the same procedure as the staging aortic lymphadenectomy in LACC without increasing surgical or postoperative complications and without delaying the start of CRT compared to single aortic lymphadenectomy.

## 1. Introduction

In 2020, 604,127 new cases of cervical cancer were diagnosed globally [1]. Despite population screening programs, a significant number of cervical cancers are detected in locally advanced stages (LACC), including IB3, IIA2-III and IVA FIGO 2018 [2]. Optimal treatment is important since a significant percentage of patients will develop recurrent or metastatic disease. For most cases with disease not amenable to therapy with curative intent, the standard of care is platinum-based chemotherapy and Bevacizumab. There is currently no standard therapy for second-line treatment, and there is no evidence that it improves survival compared with the best supportive care, with response rates of 10%. However, women in this situation are often symptomatic and relatively young, and treatment is worthwhile. There is increasing interest in the role of immunotherapy, particularly as the causative role of human papilloma virus (HPV) is well established. Vaccine-based therapies, adoptive T-cell therapy, immune-modulating agents and immune-checkpoint inhibitors are under evaluation. [3,4] As has been proven, lymph node (LN) metastasis is an important prognostic factor which has led to the addition of two new subgroups to the FIGO classification in 2018. [2] For the study of LN involvement, European guidelines provide the option of performing a histological study by means of aortic lymphadenectomy or imaging study, mainly by magnetic resonance imaging (MRI) and/or positron emission tomography (PET-CT) [5]. Accurate knowledge of aortic LN involvement is important, as it determines treatment with extended-field radiation therapy (EFRT) [5]. Far less studied is the possibility of excision of radiologically enlarged pelvic nodes in the same surgical act with the aim of confirming the pathological diagnosis and facilitating the sterilization of the pelvic field for radiotherapy (RT). In recurrent disease, surgical resection or RT may potentially be curative for selected women. The management of recurrences depends mainly on previous therapeutic approaches and on the site and extent of the disease. Pelvic exenteration usually represents the only curative treatment for patients with central pelvic failure who have previously undergone RT [4].

The first studies that proposed performing pelvic lymph node debulking in LACC date from 1989–1998 [6,7,8]. In 2008, Querleu and Morrow classified lymphadenectomy into 3 types: diagnostic (includes random sampling or sentinel technique), systematic and debulking [9], and they described the laparoscopic aortic lymphadenectomy and pelvic debulking technique used today [10]. The European guidelines contemplate the possibility of performing the excision of voluminous pelvic lymph node (PLN) detected in PET-CT/CT/MRI with the intention of debulking in LACC, individually and regardless of the performance of aortic lymphadenectomy [5]. Nonetheless, this practice has not been widely incorporated in clinical practice, either because these nodes are included in the field of RT, or because of the difficulty of the surgical technique, the increase in operative time, or complications. However, advances in minimally invasive surgery (MIS) mean that these premises may not be true, since the complications of performing pelvic LN debulking in the same operative act of aortic lymphadenectomy are probably less than those described previously [7,8,11], while it is the only procedure that allows an exact knowledge of the presence of LN involvement.

The option of surgical resection of bulky LN has been proposed, and the outcomes of interest include feasibility, morbidity and survival. So far, a survival benefit hasn’t been proven, but studies are scarce. Our group has previously evaluated the oncological effect of pelvic LN debulking in the largest retrospective series published to date with 381 patients, and we were not able to demonstrate a positive effect on survival. However, it allowed to confirmation of LN disease in 43.3% of patients with IIIC1r, avoiding false positives in imaging tests and the overtreatment with pelvic irradiation boosts. Also, chemoradiotherapy (CRT)-related toxicity in the study group was significantly lower [12]. Furthermore, in the randomized study of Uterus-11 comparing aortic and pelvic lymphadenectomy staging to imaging previous to CRT in LACC, surgery demonstrated greater cancer-specific survival, without benefits on overall survival (OS) and disease-free survival (DFS) [13].

In contrast to existing studies evaluating systematic pelvic lymphadenectomy after aortic lymphadenectomy [14,15], this study focuses on the PLN debulking technique. The aim of this study was to assess the feasibility, perioperative morbidity and time to start of RT for pelvic lymph node debulking associated with laparoscopic aortic lymphadenectomy for lymph node staging in LACC by comparing it with a control group of only aortic lymphadenectomy.

## 2. Materials and Methods

### 2.1. Study Design and Participants

This was a multicenter retrospective comparative cohort study of patients with LACC stages IIIC1r and higher (FIGO 2018) [1], carried out at eleven Spanish gynecological cancer referral hospitals. The inclusion criteria were: (1) histological confirmation of squamous, adeno-squamous, adenocarcinoma or undifferentiated cervical cancer; (2) evaluation of the tumor and LN extension with imaging tests (PET-CT and/or MRI: MRI was the gold standard in the 2000s and PET-CT has been incorporated into clinical practice since 2009); (3) suspicion of pelvic nodal involvement in imaging tests (stage IIIC1r and higher); and (4) treatment with CRT (pelvic irradiation field). Patients were recruited between 2000 and 2016, although not all hospitals included patients from the beginning of the study, as they did not introduce the surgical technique until 2010. Patients older than 80 years and/or with Eastern Cooperative Oncology Group (ECOG) ≥ 2 were excluded, as well as those staged exclusively by imaging tests, with the presence of carcinomatosis at the time of laparoscopy, and with lack of relevant data or loss to follow-up during the first two years. Data from patients were obtained retrospectively from their medical records. The clinical and histopathological characteristics, type of treatment, and perioperative morbidity of the two patient cohorts with pelvic LN involvement by imaging tests were compared. Those who underwent surgical staging by extraperitoneal laparoscopic aortic lymphadenectomy and pelvic lymph node debulking (group 1), and those with aortic lymphadenectomy without pelvic debulking (group 2).

The study was approved by the Clinical Research Ethics Committee of Hospital Universitari Vall d’Hebron (Study protocol 159/2015) as the reference center and by the Institutional Review Boards of the participating hospitals. Written informed consent was obtained from all patients.

### 2.2. Staging of Lymph Node Involvement by Imaging Test

Imaging tests were reviewed by a single radiologist in each participating hospital. Enlarged pelvic nodes were considered as those larger than 1 cm in their short diameter by MRI. In the PET-CT, the areas of increased uptake of the 18F-fluorodeoxyglucose (FDG) tracer were qualitatively evaluated: positive nodes were considered as those with increased uptake compared to the background. Enlarged LN identified to the bifurcation of the common iliac artery were considered as pelvic nodes [12,16,17].

### 2.3. Surgical Staging Procedure

Aortic lymphadenectomy was performed by extraperitoneal laparoscopy or robot-assisted, from the left side of the patient, following the technique described by Querleu [18] and modified by our group [19] and previously described elsewhere. [16,17] Lymph nodes from the interaortocaval, inferior precavo, pre-aortic and left para-aortic spaces were included up to the left renal vein, bilateral common iliac up to the bifurcation (as caudal limit) and to the psoas muscle laterally.

Pelvic lymph node debulking: in group 1, pelvic lymph node debulking was performed after extraperitoneal aortic lymphadenectomy if there were enlarged LN in the preoperative imaging tests. Pelvic LN excision was directed by imaging techniques and only pathological nodes were removed. In addition to imaging enlarged nodes, intraoperatively enlarged nodes were also removed. A systematic lymphadenectomy was not performed in any case. The LN tissue included in the procedure was the obturator fossa, external iliac regions and the lower half of the common iliac vessels.

At the beginning of the study, after completing the extraperitoneal aortic lymphadenectomy, the approach was changed to transperitoneal. In 2008, Querleu described pelvic debulking using the extraperitoneal route [10], and from then on this approach was carried out in cases where it was feasible depending on the location and access to the nodes. The technique used, with the creation of the presacral space for the exposure of the iliac vessels, has been previously described by our group [20].

Lymph node dissection was carried out using blunt dissection, monopolar and bipolar energy, and vessel sealing devices. Careful dissection was performed, and all excised LN were placed without fragmentation in endoscopic bags to avoid potential tumor spread [20]. To prevent lymphocele formation, drains were used when the approach was totally extraperitoneal, with the aim of not communicating the extraperitoneal cavity with the abdominal cavity in cases of suspected pelvic LN involvement. As of 2008, the transperitoneal approach incorporated marsupialization, which involves an opening of the peritoneum of the caudal part of the left paracolic gutter to favor lymphatic drainage [21].

The LN were carefully separated from adipose tissue by the pathologist, divided in multiple sections, and embedded in paraffin blocks. Sections of 5 μm were stained with routine hematoxylin eosin. Immunohistochemistry staining was required in one instance to identify nodal metastasis [12]. Extracapsular extension was defined as the infiltration of the capsule by tumor cells or the presence of these in the periganglionic adipose tissue.

### 2.4. Complications

Perioperative outcomes evaluated included surgical time from skin incision to closure, blood loss (estimated by the surgeon based on the blood in the aspiration reservoirs), number of nodes removed, length of hospital stay and intra- and postoperative complications. Intraoperative complications were described according to the site and type of complication. The Clavien-Dindo classification was used to categorize postoperative complications according to severity [22], and they were divided into early (≤30 days after surgery) and late (>30 days). Major complications were considered to be grade IIIB or higher. Likewise, it was specified whether they were related to the pelvic debulking procedure or aortic lymphadenectomy, and described according to the affected organ. The management of complications and the need for hospital readmission was also collected. Finally, a delay in starting CRT was considered if this took longer than 45 days or when radiation simulation had been already performed to initiate treatment but, as a result of complications from surgery, RT could not be started.

### 2.5. Chemoradiation Treatment 

All patients received external RT up to a total dose of 45 Gray (Gy) in 25 fractions in five weeks concomitantly with cisplatin 40 mg/m^2^ weekly. All patients received brachytherapy (BT) 30–35 Gy. An additional boost was administered up to a maximum of 85 Gy in cases of histologically confirmed positive nodes in group 1 or suspected pelvic nodes by imaging in group 2. All patients in which aortic LN involvement was histologically confirmed received EFRT [12,16,17].

### 2.6. Statistical Analysis

Categorical variables are expressed as frequencies and percentages, and quantitative variables as median and interquartile range (IQR) (25–75th percentile). Continuous variables were compared using a Student’s *t* test or a Mann-Whitney U test. For groups with continuous data, the analysis of variance (ANOVA) or Kruskal-Wallis test for groups of three or more continuous data were used. Categorical variables were compared using the chi-square test or Fisher’s exact test when the expected frequencies are less than five in any case. Survival curves were described by the Kaplan-Meier method and then compared using the log-rank test. Statistical significance was set at *p* < 0.05. R statistical software (version 3.6.1) was used for data analysis.

## 3. Results

**Figure 1:**
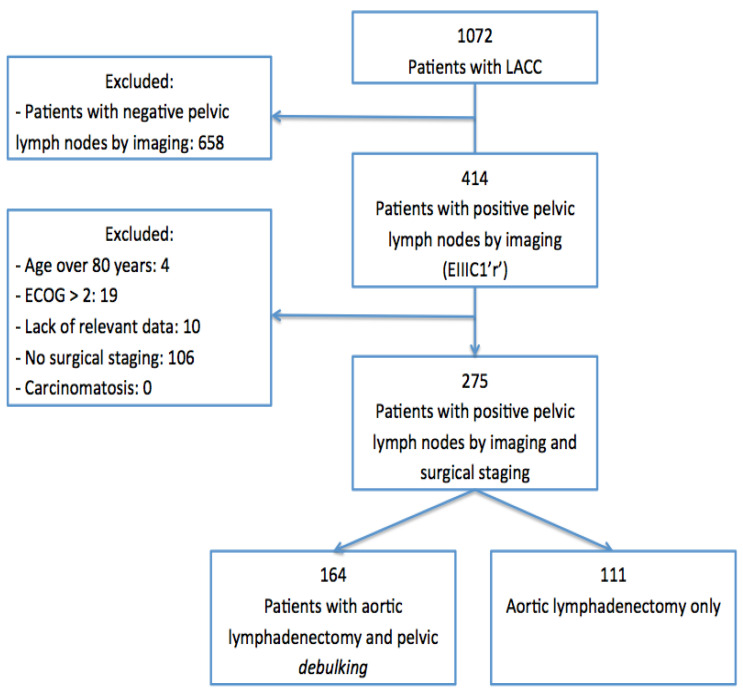
Flow chart of the study.

Out of 1072 patients with LACC, 414 had suspected pelvic LN involvement in imaging tests (stage IIIC1r), and 275 were finally included in the study: 164 patients in group 1 and 111 patients in group 2. The study flow diagram is shown in Figure 1. Patient baseline characteristics, diagnostic imaging tests and the treatment strategy for each group are shown in Table 1.

### 3.1. Surgical Staging

Surgery was performed by conventional laparoscopy in 93.9% of cases in group 1 and 96.4% in group 2, while the remaining cases in each group were performed by robot-assisted surgery. Excision of all the lymph nodes was possible in all patients except for one in group 1 in whom it was not technically feasible due to bone infiltration and three patients in group 2 who presented unresectable adenopathic conglomerates. Marsupialization was performed in 73.1% of patients in group 1 and in 66.6% in group 2. In group 1 the surgery lasted a median of 82 min longer than in group 2 (215 min [IQR 170–240] vs. 132.5 min [IQR 113.8–150]; *p* < 0.001). Intraoperative bleeding was equal between the groups (40 mL in group 1 [IQR 20, 40] vs. 50 mL [IQR 32.5, 147.5] in group 2; *p* 0.05), and the median length of hospital stay was two days in both groups. 

A median of 8.5 nodes were extracted (IQR 3–13) in patients undergoing a pelvic LN debulking procedure, with 42.3% being positive. A total of 50 (70.4%) of the metastatic pelvic nodes were larger than 5 mm, with a median size of 13 mm [IQR 8–18], and 42% had extracapsular involvement. 

Other data regarding surgery are shown in Table 2.

### 3.2. Surgery-Related Complications

Intraoperative complications occurred in six patients (3.7%) in group 1 and three patients (2.7%) in group 2. None of the complications were related to the pelvic debulking procedure. In order of frequency, they were vascular and urological complications with two external iliac vein injuries, one renal vein injury and three unspecified, as well as two ureteral sections, and one accidental opening of the peritoneum at the time of laparoscopic entry.

Early postoperative complications were observed in 13 cases (8%) in group 1, three of them related to pelvic debulking (two local infections and a pelvic lymphocele), and in seven cases in group 2 (6.3%). Lymphatic complications were the most frequent type, with seven cases of lymphocele (2.5%) and a case of lymphedema and chylous ascites (0.3%). No differences were observed between groups in terms of the severity of complications according to the Clavien-Dindo classification [22], the way of resolution of the complication or the need for hospital readmission.

As for late postoperative complications, these occurred in 10 patients (6.1%) in group 1 (two occurred in relation to pelvic lymph node debulking with the development of lymphedema), and three (2.7%) in group 2. Complications were lymphatic in eight out of 13 cases (2.9%): four lymphedema and four lymphoceles. There were no differences between groups regarding the severity of complications according to the Clavien-Dindo classification [22], nor for the way of resolution of the complication. There were more hospital readmissions in group 1 compared to group 2 (3.1% vs. 0.9%, *p* 0.047), one of which was in the ICU (unspecified reason).

A significantly shorter time from diagnosis to the start of RT was observed in group 1 compared to group 2 (median 61 days vs. 70.5 days; *p* 0.006), and also between surgery and the start of RT (29 days in group 1 vs. 39 in group 2; *p* < 0.001). However, there were no differences in terms of delay in the initiation of treatment due to surgical complications (0.6% in group 1 and 0.9% in group 2).

The detailed description of the complications is shown in Table 3. 

## 4. Discussion

In our study, performing laparoscopic pelvic lymph node debulking in the same surgical act as extraperitoneal aortic lymphadenectomy for staging in LACC proved to be feasible and did not lead to an increase in surgical morbidity or a delay in the initiation of CRT treatment, in comparison with those patients who only underwent aortic lymphadenectomy. This procedure allowed us to confirm LN metastatic disease in a significant percentage of patients with suspected pathological pelvic lymph nodes in imaging tests, selecting those patients who truly required an additional pelvic RT boost.

### 4.1. Surgical Technique

Given that in debulking surgery the nodes are usually of considerable size and excision can be difficult, the results of the current study should be carefully compared to other studies evaluating systematic pelvic lymphadenectomy [13,14], as they comprise different techniques. When there is extracapsular involvement, debulking can be technically difficult, and this was already reported by Querleu who considered unresectable lymph nodes to be those measuring >3 cm and those with extracapsular involvement [10]. In addition, Zighelzoim et al. stated that the ability to completely resect suspected metastatic LN at the time of laparotomic extraperitoneal LN dissection is associated with the size and location of the largest node [23]. Toyama et al. goes further and proposes performing pelvic LN debulking if the following conditions are met: the absence of distant metastases, the primary cervical tumor can be controlled by CRT or RH plus adjuvant CRT, and the bulky lymph nodes are not involving major blood vessels and are not accompanied by bone infiltration [24]. Nonetheless, in our study, excision of bulky nodes was feasible in the majority of cases. Thanks to the magnification of the image with laparoscopy, the experience of the surgeon, and through careful dissection and the use of instruments such as vessel sealants, our experience shows that it is possible to remove the entire node with an intact capsule, regardless of the extra or transperitoneal approach. The extraperitoneal approach is a relatively recent technique for approaching the aortic area, but the acquired learning curve of surgeons has made it possible to perform pelvic LN debulking through this same access without the need to change to the transperitoneal route. This could reduce the surgical time due to the change of approach and the number of laparoscopic incisions. In addition, the extraperitoneal route reduces the de novo adhesion compared to the transperitoneal route [25,26,27]. Thus, we believe that the extraperitoneal approach should be the technique for PLN debulking in LACC whenever possible. 

In our study, the surgical time was longer in the debulking group. Although this result was expected, it did not have a relevant clinical impact since it was not associated with a longer length of hospital stay or an increase in intraoperative bleeding. In comparison, Tozzi et al. reported 22 cases of PLN debulking in LACC, 12 of them associated with transperitoneal laparoscopic aortic lymphadenectomy, achieving complete excision in all cases except in one patient with a 3 cm lymph node attached to the lumbosacral trunk [28]. Table 4 summarizes the available reports on the feasibility and morbidity of lymph node debulking surgery in cervical cancer. 

### 4.2. Surgical Morbidity

An argument against performing this surgery is the hypothetical increase in complications, especially with the laparotomic approach. However, with advances in MIS and the benefits of the extraperitoneal approach [25,26,27], morbidity is now minimal. In the present study, intraoperative complications were low and similar between groups. Tozzi and Querleu did not report intraoperative complications in their retrospective series of laparoscopic pelvic debulking in LACC, although they had a limited sample size [10,28]. In the first analysis of the randomized study Uterus-11 on LN staging using transperitoneal laparoscopic aortic and pelvic lymphadenectomy in LACC in 123 patients, Köhler et al. described a 1.6% intraoperative complication rate in the form of vascular lesions [30]. For the same surgery, Mezquita et al. described 5.9% of intraoperative complications (one lesion in the vena cava, one lesion in the obturator nerve, and two ureteral lesions) [15]. We consider that our complications, mainly vascular and urological and a complication at entry, are inherent to the risk of any surgical act and in this case to the excision of metastatic LN, which often implies adhesions to the adjacent structures, possibly vascular-nervous. Additionally, there were no significant differences in early or late postoperative complications between the groups. We only observed a minimal non-significant increase in group 1, which could be attributable to the surgical difficulty of excising bulky nodes. However, the results are consistent with those reported by others authors, with postoperative complication rates ranging between 7.6% and 10.4% [13,28] (Table 4). 

The most frequent complications were lymphatic, especially the development of lymphoceles. These results are comparable to those described by others, with rates of lymphocele and lymphedema around 3%, and chylous ascites of 0.8–1.5% [15,30]. In fact, Mezquita highlighted that adding pelvic lymphadenectomy to the aortic excision increases the risk of lymphedema [15]. Given that the excision was guided by imaging techniques (stage IIIC1r), the slight increase in lymphatic complications in the debulking group could be explained by an increased lymphorrhea when performing the excision of metastatic nodes with the rupture of the dilated lymphatic canaliculi. In order to minimize these complications given their impact on the quality of life of patients, maneuvers were incorporated for the prevention of lymphoceles. For the transperitoneal approach, a large marsupialization was performed and a drain was inserted in order to avoid the accumulation of lymph fluid in the abdominal cavity, and drains were also used in the extraperitoneal route for the same reason. As these preventive maneuvers were not performed from the beginning of the study period, it is possible that the results in terms of lymphatic complications have improved in recent years.

### 4.3. Delay in CRT Treatment

Another concern regarding staging surgery in LACC is the possibility that it may delay the start of CRT treatment and negatively affect the disease prognosis. [31] In our study, no such delay was observed, with an even shorter time from surgery to the start of RT in the study group. These results are in line with others in the literature, with reported times of 12–28 days between surgery and RT [15,16,28,30], and could be explained by the advantages in terms of rapid recovery from MIS and the experience of the surgical team. However, we believe that these results can be improved, and efforts should be made to further reduce the time between surgery and the start of RT. That is why we emphasize the need to centralize the management and treatment of these patients in gynecological oncology referral hospitals. 

### 4.4. Strengths and Limitations

To our knowledge, this study includes the largest number of patients that specifically assessed the feasibility and morbidity of performing pelvic LN debulking associated with laparoscopic aortic lymphadenectomy in LACC and comparing it with a control group. It is noteworthy that this is the first study to specifically evaluate the delay produced by the addition of pelvic debulking to staging surgery, when compared with a control group with only aortic lymphadenectomy. Despite this, we recognize important limitations, including its retrospective and multicenter design without a unified action protocol. This meant that the completion of the pelvic debulking was performed according to the criteria of each center. Nevertheless, only two of the participating centers performed the described surgical technique, which reduces the possible heterogeneity. And regarding the heterogeneity, this study lacked a centralized review of imaging and pathology results. Additionally, the pelvic debulking approach was not differentiated between extra or transperitoneal, as it was not a variable recorded in many records. Therefore, we cannot compare both approaches. Lastly, the study collection spanned 16 years, and not all cases were evaluated by PET-CT, which is currently considered the gold-standard. The reason for this is that some hospitals only began performing PET-CT and staging aortic lymphadenectomy in 2010.

### 4.5. Implications for Practice and Future Research

Laparoscopic pelvic LN debulking has the dual objective of staging and debulking. First, it allows the identification of the metastatic lymph node involvement, thereby avoiding false positives and negative results of imaging tests. Secondly, it facilitates the local action of RT by reducing the tumor burden. Few studies have evaluated this procedure and its practice has not been extended, despite the benefits it can offer to patients with LACC. In our study we demonstrated that it is a feasible and safe procedure that can be implemented in centers with advanced management in gynecological laparoscopic surgery. The European Society of Gynecological Oncology recently published a set of surgical-related quality indicators that can be a major asset for centers to improve the quality of surgical treatment of cervical cancer [32] and accreditation programs have been developed in other gynecological tumors. Regarding pelvic lymph node debulking surgery, further research is required to assess its benefits in terms of survival.

## 5. Conclusions

In conclusion, it is feasible to perform laparoscopic pelvic node debulking via the extraperitoneal or transperitoneal route in the same surgical procedure as the staging of the aortic lymphadenectomy in LACC without increasing surgical complications. This procedure allows us to know the lymph node status of the patients without increasing perioperative morbidity or delaying the start of treatment with CRT. Hence, pelvic lymph node debulking is a feasible and safe option that should be considered individually in trained centers, since it is a surgery that requires training and advanced management in laparoscopic pelvic surgery.

## Figures and Tables

**Table 1:** Baseline characteristics, imaging tests and treatment of both groups of patients.

Variables	Group 1 ^a^ (164)	Group 2 ^b^ (111)	All (275)	*p* Value
Age at diagnosis, median (IQR) ^c^	48.5 (40, 58)	49 (41, 55)	49 (40.5, 57)	
BMI ^d^, kg/m^2^, median (IQR)	26 (22.2, 29.5)	25.3 (21.7, 29.4)	25.8 (22, 29.5)	
ECOG ^e^ at diagnosis, *n* (%):				
0	129 (78.6)	93 (83.7)	222 (80.7)	
1	35 (21.3)	18 (16.2)	53 (19.2)	
Histological type, *n* (%):				
Squamous	132 (80.4)	99 (89.1)	231 (84)	
Adenocarcinoma	27 (16.4)	9 (8.1)	36 (13.1)	
Histological grade, *n* (%):				
G1: well-differentiated	6 (3.6)	6 (5.4)	12 (4.3)	
G2: moderately-diff.	41 (25)	26 (23.4)	67 (24.3)	
G3: poorly-differentiated	50 (30.4)	33 (29.7)	83 (30.1)	
Not available	67 (40.8)	45 (40.5)	112 (40.7)	
Imaging tests, *n* (%):				
MRI ^f^	156 (95.1)	108 (97.3)	264 (96)	
Positive pelvic lymph nodes	99 (60.3)	96 (86.4)	195 (70.9)	<0.001
Positive aortic lymph nodes	15 (9.1)	2 (1.8)	17 (6.1)	0.023
Positive pelvic and/or aortic	110 (67.1)	96 (86.4)	206 (74.9)	<0.001
PET-CT ^g^	51 (31.1)	56 (50.4)	107 (38.9)	0.002
Positive pelvic lymph nodes	35 (21.3)	46 (41.4)	81 (20.4)	
Positive aortic lymph nodes	10 (6.1)	6 (5.4)	16 (5.8)	
Positive pelvic and/or aortic	37 (22.5)	46 (41.4)	83 (30.1)	
Radiotherapy, *n* (%)	164 (100)	111 (100)	275 (100)	
Chemotherapy, *n* (%)	148 (90.2)	100 (90.1)	248 (90.2)	
Brachytherapy, *n* (%)	19 (11.6)	25 (22.5)	44 (16)	0.033
Acute toxicity, *n* (%)	15 (9.1)	23 (20.7)	38 (13.8)	0.013
Late toxicity, *n* (%)	26 (22.2, 29.5)	25.3 (21.7, 29.4)	25.8 (22, 29.5)	

^a^ Group 1: aortic lymphadenectomy and pelvic debulking; ^b^ Group 2: aortic lymphadenectomy; ^c^ IQR: interquartile (25–75th percentile); ^d^ BMI: body mass index; ^e^ ECOG: Eastern Cooperative Oncology Group; ^f^ MRI: magnetic resonance imaging; ^g^ PET-CT: positron emission tomography-computed tomography.

**Table 2:** Description of the surgery in both groups of patients.

Variables	Group 1 ^a^ (164)	Group 2 ^b^ (111)	All (275)	*p* Value
Type of laparoscopy, *n* (%):				
Conventional	154 (93.9)	107 (96.4)	261 (94.9)	
Robot-assisted	9 (5.5)	4 (3.6)	13 (4.7)	
Marsupialization, *n* (%):				
Yes	120 (73.1)	74 (66.6)	194 (70.5)	
No	37 (22.5)	25 (22.5)	62 (22.5)	
Drain, *n* (%)	40 (24.3)	15 (13.5)	55 (20)	0.043
Transfusion, *n* (%)	7 (4.2)	6 (5.4)	13 (4.7)	
Intraoperative bleeding (mL), median (IQR) ^c^	40 (20,40)	50 (32.5, 147.5)	50 (20, 78.8)	0.05
Operative time (min), median (IQR)	215 (170, 240)	132.5 (113.8, 150)	180 (130, 240)	<0.001
Hospital stay (days), median (IQR)	2 (2, 3)	2 (1,3)	2 (2, 3)	0.012
Pelvic lymph node removed, median (IQR)	8.5 (3, 13)	--	8.5 (3, 13)	--
Positive pelvic lymph nodes	71 (43.3)	--	--	--
Negative pelvic lymph nodes	93 (56.7)	--	--	--
Size of affected pelvic lymph nodes, *n* (%)				
≤5 mm	10 (14)	--	--	--
>5 mm	50 (70.4)	--	--	--
Unknown	11 (15.5)	--	--	--
Size of affected pelvic lymph nodes (mm), median (IQR)	13 (8, 19)	--	--	--
Extracapsular involvement of pelvic nodes	21 (42)	--	--	--
Aortic lymph node removed, median (IQR)	12 (9, 19)	14.5 (10.2, 19)	13 (9, 19)	
Positive aortic lymph nodes	40 (24.4)	33 (29.7)	73 (26.5)	
Negative aortic lymph nodes	124 (75.6)	78 (70.3)	202 (73.4)	

^a^ Group 1: aortic lymphadenectomy and pelvic debulking; ^b^ Group 2: aortic lymphadenectomy; ^c^ IQR: interquartile (25–75th percentile).

**Table 3:** Early and late intra- and postoperative complications for both groups. Severity graded according to Dindo-Clavien classification [20].

Variable	Group 1 ^a^ (164)	Group 2 ^b^ (111)	All (275)	*p* Value
Intraoperative complications, *n* (%)	6 (3.7)	3 (2.7)	9 (3.3)	
Debulking-related	0 (0)	--	--	--
Aortic lymphadenectomy-related: ureteral injury and iliac vein injury	1 (0.6)	1 (0.9)	2 (0.7)	
Site of complication:				
Abdominal wall (peritoneum opening during entry)	1 (0.6)	0 (0)	1 (0.4)	
Urological	1 (0.6)	1 (0.9)	2 (0.7)	
Vascular	4 (2.4)	2 (1.8)	6 (2.1)	
Early postoperative complications, *n* (%)	13 (8)	7 (6.3)	20 (7.3)	
Grade I	3 (1.8)	1 (0.9)	4 (1.5)	
Grade II	6 (3.6)	1 (0.9)	7 (2.5)	
Grade III:	4 (2.4)	2 (1.8)	6 (2.2)	
Grade IIIA	2 (1.2)	2 (1.8)	4 (1.5)	
Grade IIIB	2 (1.2)	0 (0)	2 (0.7)	
Grade IV	0 (0)	1 (0.9)	1 (0.4)	
Debulking-related:	3 (1.8)	--	--	--
Lymphocele	1 (0.6)	--	--	
Local infection	2 (1.2)	--	--	
Aortic lymphadenectomy-related:	4 (2.4)	4 (3.6)	8 (2.9)	
Retroperitoneal hematoma	0 (0)	1 (0.9)	1 (0.3)	
Lymphocele (7), lymphedema and chylous ascites (1)	4 (2.4)	4 (3.6)	8 (2.9)	
Other complications:				
Surgical wound infection and sepsis	0 (0)	1 (0.9)	1 (0.3)	
Deep venous thrombosis	1 (0.6)	0 (0)	1 (0.3)	
Way of resolution of the complication:				
Laparoscopy	2 (1.2)	0 (0)	2 (0.7)	
Laparotomy	0 (0)	0 (0)	0 (0)	
Interventional radiology	1 (0.6)	2 (1.8)	3 (1.1)	
Others	10 (6.0)	3 (2.7)	13 (4.7)	
Readmission due to complication:	4 (2.4)	1 (0.9)	5 (1.8)	
Normal hospitalization	4 (2.4)	1 (0.9)	5 (1.8)	
Admission to ICU	0 (0)	0 (0)	0 (0)	
Late postoperative complications, *n* (%)	10 (6.1)	3 (2.7)	13 (4.7)	
Grade I	2 (1.2)	0 (0)	2 (0.7)	
Grade II	1 (0.6)	1 (0.9)	2 (0.7)	
Grade III:	7 (4.3)	1 (0.9)	8 (2.9)	
Grade IIIA	3 (1.8)	1 (0.9)	4 (1.4)	
Grade IIIB	4 (2.4)	0 (0)	4 (1.4)	
Grade IV	0 (0)	1 (0.9)	1 (0.4)	
Debulking-related:	2 (1.2)	--	--	--
Lymphedema	2 (1.2)	--	--	--
Aortic lymphadenectomy-related:	9 (5.5)	3 (2.7)	12 (4.4)	
Lymphocele (4), lymphedema (x2), umbilical hernia (1)	5 (3.1)	3 (2.7)	8 (2.9)	
Other complications:				
Deep venous thrombosis	1 (0.6)	0 (0)	1 (0.3)	
Way of resolution of the complication:				
Laparoscopy	2 (1.2)	0 (0)	2 (0.7)	
Laparotomy	2 (1.2)	0 (0)	2 (0.7)	
Interventional radiology	3 (1.8)	1 (0.9)	4 (1.4)	
Others	3 (1.8)	1 (0.9)	4 (1.4)	
Readmission due to complication:	5 (3.1)	1 (0.9)	5 (1.8)	0.047
Hospitalization floor	4 (2.4)	1 (0.9)	4 (1.4)	
ICU ^c^	1 (0.6)	0 (0)	1 (0.4)	
Delay in the start of CRT ^d^, *n* (%)	1 (0.6)	1 (0.9)	2 (0.7)	
Time from diagnosis to RT ^e^ (days), median (IQR) ^f^	61 (47,80)	70.5 (54,92)	63 (50,88)	0.006
Time from surgery to RT (days), median (IQR)	29 (20,40)	39 (23,53)	31 (31.2, 45)	<0.001

^a^ Group 1: aortic lymphadenectomy and pelvic debulking; ^b^ Group 2: aortic lymphadenectomy; ^c^ ICU: intensive care unit; ^d^ CRT: chemoradiotherapy; ^e^ RT: radiotherapy; ^f^ IQR: interquartile (25–75th percentile).

**Table 4:** Summary of studies that report complications related to lymph node debulking in cervical cancer.

Author (Year)	Period of Study and Design	Total of Patients	FIGO ^a^ 2008 Stage	Surgical Approach	Primary Treatment	% of Unresectable Nodes	Intraoperative Complications	Postoperative Complications
Hacker et al. (1995) [7]	1987–1992 Retrospective	34	IB-IIIB	Laparotomic extraperitoneal	RT ^d^ ± CT ^e^	0	2.9%: external iliac vein injury	11.8% Acute: - Necrotizing fasciitis - Cytomegalovirus hepatitis - Infected lymphocysts 18% Long-term: - Radiation enteritis - Ischemic bowel
Cosin et al. (1998) [8]	1978–1990 Retrospective	266	IB-IV	Laparotomic extraperitoneal	RT ^d^ ± BT ^c^ ±CT ^e^	7.5% (adherence or invasion of vascular or nervous structures)	Not reported	19.9% lymphoceles 18.4% lymphedema 10.5% complications requiring significant medicanl intervention or surgery 1.1% CRT-related deaths
Marnitz et al. (2005) [29]	1994–2003 Prospective	84	IB1-IVB	Laparoscopic transperitoneal	CRT ^b^ + BT ^c^	Not reported	0	15.5% limphoceles > 5 cm
Zighelboim et al. (2006) [23]	1990–2004 Retrospective	104	IA-IVA	Laparotomic	RT ^d^/CRT ^b^	15% (vascular involvement 43.5%, infiltration into the bone 19%, neural invasion 12.5%, unknown 25%)	6%: vascular injuries	27%: - Anemia 9.6% - Wound complication 7.7% - Pneumonia 2.8% - Lymphocyst 1.9% - Fever of unknown origin 3.8% - Radiation enteritis 0.9%
Querleu et al. (2008) [10]	Not reported Prospective	8	IB1-IIIA	Laparoscopic extraperitoneal	CRT ^b^	0 (excluded patients with nodes > 3 cm)	0	12.5%: Transient and partial motor defect in the territory of obturator nerve
Tozzi et al. (2009) [28]	2006–2008 Prospective	22	IB2-IIIB	Laparoscopic transperitoneal	CRT ^b^ + BT ^c^	4.6% (node adjacent to the lumbosacral trunk)	0	9%: - Thermal injury of the sciatic root - Chylous ascites
Our study	2000–2016 Retrospective	275 (164 debulking group)	IIIC1r-IVA (FIGO ^a^ 2018)	Laparoscopic trans/extraperitoneal	CRT ^b^ + BT ^c^	0.6% (bone infiltration)	3.6% (four vascular injuries, one ureteral injury, one accidental opening on the peritoneum)	8% Early: two local infections, four lymphoceles, one retroperitoneal hematoma, one DVT ^f^, five unspecified abdominal wall complications. 6.1% Late complications

^a^ FIGO: International Federation of Gynecology and Obstetrics; ^b^ CRT: chemoradiotherapy; ^c^ BT: brachytherapy; ^d^ RT: radiotherapy; ^e^ CT: chemotherapy; ^f^ DVT: deep venous thrombosis.

## Data Availability

The data presented in this study are available in this article.
